# Inhibition-based biosensor for cyanide detection – a preliminary study

**DOI:** 10.1080/07853890.2021.1896915

**Published:** 2021-09-28

**Authors:** Ana R. Coelho, Tiago Monteiro, Ana S. Viana, M. Gabriela Almeida

**Affiliations:** a Fac. de Ciências, Universidade de Lisboa, Lisboa, Portugal; b Fac. Ciências e Tecnologia – NOVA, Caparica, Portugal; c Centro de Investigação Interdisciplinar Egas Moniz (CiiEM), Egas Moniz Cooperativa de Ensino Superior, Caparica, Portugal

## Abstract

**Introduction:**

The acute toxicity of cyanide along with its continue industrial use makes this substance of environmental concern [1]. Titration, spectrophotometry and ISE are the standard detection methods. However, they are complex and need sample pre-treatment [2]. To overcome these, another approach is using biosensors. To this end, we developed a disposable inhibition-based biosensor with a multi-heme nitrite reductase (c*c*NiR) coupled to graphite leads.

**Materials and methods:**

Electrochemical measurements were carried out in a conventional electrochemical cell, composed by a three-electrode system. The reference was an Ag/AgCl electrode, and the counter electrode was a Pt wire. The working electrode (WE) was in-house made using a graphite lead with the c*c*NiR (from bacteria D. *desulfuricans* ATCC 27774; stored in 0.05 M phosphate buffer, pH 7.6) immobilised by drop cast at the WE surface. The electrochemical technique used was square wave voltammetry. Electrochemical cells contained 0.1 M KCl in 0.1 M Tris–HCl buffer (pH 7.6) as supporting electrolyte. Dissolved oxygen was removed by a biochemical system (GOx, catalase and glucose).

**Discussion and conclusions:**

In Figure 1 we can observe the decrease in catalytic activity due to the presence of cyanide. The biosensor dynamic range comprises the maximum value imposed by the European Union, 1.92 µM (98/83/EC directive), which does not happen with other cyanide biosensors [3]. Furthermore, a graphite lead cost 0.35€ and each of them can be split, allowing a very low-cost biosensor. Given that enzyme inhibition is reversible [4], the sensor can be used more than one time, if we optimise the enzyme immobilisation method.

**Figure 1.**
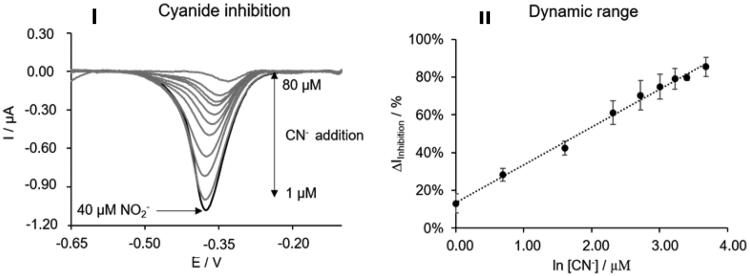
(I) Square wave voltammograms with baseline correction of one of the WE used in the study. Enzymatic inhibition by cyanide among 1–80 µM in the presence of 40 µM of nitrite. (II) Calibration curve (*y* = 0.200*x* + 0.135; *R*^2^ = 0.995) for CN^-^ quantification, with the dynamic range 1 to 40 µM. Results are expressed as triplicate average with the corresponding standard deviation.
